# Addition of Vitamin C Does Not Decrease Neuron-Specific Enolase Levels in Adult Survivors of Cardiac Arrest—Results of a Randomized Trial

**DOI:** 10.3390/medicina60010103

**Published:** 2024-01-05

**Authors:** Matevž Privšek, Matej Strnad, Andrej Markota

**Affiliations:** 1 Emergency Medical Services, Healthcare Centre Dr. Adolf Drolc, Cesta Proletarskih Brigad 21, 2000 Maribor, Slovenia; matej.strnad76@gmail.com; 2 Emergency Department, University Medical Centre Maribor, Ljubljanska Ulica 5, 2000 Maribor, Slovenia; 3 Department of Emergency Medicine, Faculty of Medicine, University of Maribor, Taborska Ulica 8, 2000 Maribor, Slovenia; 4 Department of Medical Intensive Care, Clinic of Internal Medicine, University Medical Centre Maribor, 2000 Maribor, Slovenia; andrej.markota@ukc-mb.si

**Keywords:** vitamin C, nervous system-specific enolase, out-of-hospital cardiac arrest, return of spontaneous circulation, survival

## Abstract

*Background and Objectives*: Survival with favorable neurologic outcomes after out-of-hospital cardiac arrest (OHCA) remains elusive. Post-cardiac arrest syndrome (PCAS) involves myocardial and neurological injury, ischemia-reperfusion response, and underlying pathology. Neurologic injury is a crucial determinant of survival and functional outcomes, with damage caused by free radicals among the responsible mechanisms. This study explores the feasibility of adding intravenous vitamin C to the treatment of OHCA survivors, aiming to mitigate PCAS. Vitamin C, a nutrient with antioxidative and free radical-scavenging properties, is often depleted in critically ill patients. *Materials and Methods*: This randomized, double-blinded trial was conducted at a tertiary-level university hospital with adult OHCA survivors. Participants received either standard care or the addition of 1.5 g of intravenous vitamin C every 12 h for eight consecutive doses. Neurologic injury was assessed using neuron-specific enolase (NSE) levels, with additional clinical and laboratory outcomes, such as enhanced neuroprognostication factors, inflammatory markers, and cardiac parameters. *Results*: NSE levels were non-significantly higher in patients who received vitamin C compared to the placebo group (55.05 µg/L [95% confidence interval (CI) 26.7–124.0] vs. 39.4 µg/L [95% CI 22.6–61.9], *p* > 0.05). Similarly, a non-significantly greater proportion of patients in the vitamin C group developed myoclonus in the first 72 h. We also observed a non-significantly shorter duration of mechanical ventilation, fewer arrhythmias, and reduced length of stay in the intensive care unit in the group of patients who received vitamin C (*p* = 0.031). However, caution is warranted in interpretation of our results due to the small number of participants. *Conclusions*: Our findings suggest that intravenous vitamin C should not be used outside of clinical trials for OHCA survivors. Due to the small sample size and conflicting results, further research is needed to determine the potential role of vitamin C in post-cardiac arrest care.

## 1. Introduction

Cardiac arrest is a life-threatening event characterized by the sudden cessation of cardiac activity and its function to perfuse organs and tissues. It remains one the most significant health issues in Europe and worldwide [1,2,3]. Despite numerous advances in the system of saving lives, which have resulted in an increased number of victims with return of spontaneous circulation (ROSC), overall survival with favorable neurological outcome remains low. Only about one-third of out-of-hospital cardiac arrest (OHCA) victims survive to hospital discharge, and of those, only about 25% are discharged with a good neurological status, characterized by a Cerebral Performance Category (CPC) of one and two [1,2].

After ROSC, post-cardiac arrest syndrome (PCAS) develops in virtually all survivors, except in those with only brief resuscitative efforts [4]. PCAS consists of four major components: post-cardiac arrest myocardial injury, post-cardiac arrest neurologic injury, systemic response to ischemia-reperfusion injury, along with pathology that caused cardiac arrest [4]. Neurologic injury is particularly crucial because, besides being the leading cause of death in patients after ROSC, it also plays a pivotal role in determining the functional outcome of patients who survive to hospital discharge [5]. Numerous intricate and intertwined mechanisms are responsible for the development of neurologic injury, with oxidative stress being one of them.

Vitamin C, also known as ascorbic acid or ascorbate, is a water-soluble molecule with diverse biological actions [6,7,8]. Its levels are often depleted in critically ill individuals, and low concentrations have been associated with worse outcomes [9,10]. Its antioxidative activity is paramount for neutralizing harmful free radicals, massively overproduced during ischemia and subsequent reperfusion [11,12,13].

Several trials in recent years have explored the possibility of intravenous vitamin C therapy and have shown promising results. Henry et al. and Huang et al. have demonstrated that intravenous vitamin C mitigates the extent of neurologic injury after an ischemic stroke and increases cerebral perfusion in animals [14,15]. Similar findings were established by Tsai et al. regarding the beneficial effects of vitamin C on injured myocardium [16,17,18].

The aim of this trial was to investigate the feasibility of adding vitamin C (ascorbic acid) to patients resuscitated from OHCA and, thereby, introduce a new treatment option for patients with PCAS.

## 2. Materials and Methods

### 2.1. Study Design

Our study was a single-center, prospective, randomized, double-blinded trial comparing the addition of intravenous vitamin C in adult survivors of out-of-hospital cardiac arrest to the current standard of care.

The trial was approved by the Slovenian Medical Ethics Committee (nr. 0120-342/2019/5) and registered on ClinicalTrials.gov (identifier NCT04563000). Written informed consent was obtain from the lawful representatives of included patients. The study was conducted in accordance with the Declaration of Helsinki.

### 2.2. Participating Patients

The trial was conducted at the medical intensive care unit (ICU) of a tertiary-level university hospital between 1 February 2022, and 30 September 2023. Adult patients admitted to the ICU following successful resuscitation from cardiac arrest were screened for eligibility by the admitting physician. Inclusion criteria were age at least 18 years, out-of-hospital cardiac arrest, comatose state (Glasgow Coma Scale (GCS) 8 or less) after ROSC, and available for the first dose of vitamin C within 6 h after ROSC. Exclusion criteria included trauma, asphyxia, drowning, and electrocution as the cause of cardiac arrest, past medical history of oxalate nephropathy, glucose-6-phosphate dehydrogenase deficiency, haemochromatosis, and pregnancy. These criteria were selected to maximize patients’ safety and tolerance to the intervention. Eligible patients were consecutively enrolled.

Participants were randomized to the intervention or to usual (standard) care. Randomization with a variable block size was performed by a computer. After obtaining consent, the enrolling physician consecutively assigned participants to the allocation table with the appropriate randomization code. The registered nurse, using the randomization code, then prepared and administered the treatment or placebo. Only the nurse who prepared the final solution for administering knew whether the solution contained vitamin C or not. This was necessary, as the use of a premade solution was not possible due to precipitation and stability concerns.

### 2.3. Emergency Medical System in Slovenia

The emergency medical system (EMS) in Slovenia comprises two types of prehospital teams: (A) ambulances with a nurse driver and a registered nurse, capable of basic life support (BLS), and (B) ambulances with a nurse driver, a registered nurse, and a physician, capable of advanced life support (ALS). The prehospital physician is either an emergency medicine specialist or a family medicine specialist with additional knowledge from advanced life support courses. Prehospital teams are dispatched to events in accordance with Healthcare Dispatch Centre protocols.

In cases of (presumed) cardiac arrests, dispatch activates the nearest ambulance team, regardless of type, as well as the nearest prehospital team with a physician. In cases of cardiac arrests outside urban areas, first responders are also activated. First responders are mostly voluntary firefighters, trained in BLS with the use of automated external defibrillator (AED).

After ROSC, the patient is transferred to the university hospital for immediate coronary angiography if signs of ST-elevation myocardial infarction are present. Otherwise, the patient is admitted to a regional hospital for further diagnostic workup in cases without clear ST-elevation myocardial infarction and is transferred later to the university hospital if needed.

### 2.4. Trial Intervention

The trial intervention consisted of administering 1.5 g of vitamin C diluted in 100 mL of 0.9% NaCl intravenously (IV) every 12 h for 8 consecutive doses. The dosage and regimen were selected based on the results of previous trials regarding the supplementation of vitamin C in critically ill patients [19,20,21]. The intervention was terminated early if oxalate nephropathy developed, as vitamin C can worsen this condition.

Participants in the control group received 100 mL of 0.9% NaCl IV in the same regimen as the intervention group. Both groups received standard care that follows the general principles of intensive care therapy for critically ill patients, at the discretion of participants’ physician.

### 2.5. Outcomes

The primary outcome was the neuron-specific enolase (NSE) serum level after the 96 h intervention period. NSE was selected as it is an established biomarker in prognostication after brain injury and, compared to other biomarkers of brain injury, is released only from injured neurons and no other cells [22,23].

Secondary outcomes included improved results in other tests used for neuroprognostication, such as reduced myoclonus in the first 72 h after ROSC, enhanced results of the electroencephalogram (EEG), and brain computed tomography (CT). Nonetheless, due to inconsistencies in EEG and additional brain CT utilization, these outcomes were subsequently excluded.

Given the systemic antioxidizing effect of vitamin C, we also hypothesized that a similar beneficial effect of vitamin C exists in other organ systems. We included lower serum C-reactive protein (CRP), procalcitonin (PCT), high-sensitivity troponin I (hsTnI), N-terminal pro b-type natriuretic peptide (NT-proBNP), lactate, and higher pH of arterial blood in other outcomes.

Clinical outcomes included a shorter duration of mechanical ventilation, more successful extubations, reduced need for arrhythmia treatment, new onset heart failure, need for percutaneous coronary intervention (PCI), and renal replacement therapy, along with secondary infection, time spent in the ICU and the hospital, and increased 30-day survival.

### 2.6. Data Collection and Statistical Analysis

Data regarding management and treatment in the ICU were collected from the hospital electronic health record (EHR), while data about cardiac arrest circumstances and resuscitation were obtained from EMS records.

NSE was sampled at least once between 48 and 96 h after cardiac arrest, and the higher value of NSE in this period was used in the analysis. Patients who remained comatose after 72 h after cardiac arrest had their first EEG and brain CT done between 72 and 120 h after admission (3rd–5th day). In cases of persisting unconsciousness, a second EEG and brain CT was performed between the 7th and 10th day after admission.

Results of CTs were descriptive and analyzed as nominal variables. The result of the first brain CT was classified as normal, slightly abnormal, moderately abnormal, and severely abnormal. Brain CT results were considered abnormal when signs of a global ischemic insult were present: generalized edema, loss of grey–white matter differentiation, and obliteration of gyri sulci. Brain CT results were analyzed and interpreted by a neuroradiologist as normal or as slight, moderate, or severe brain damage.

A full clinical exam was performed at least twice daily, with emphasis on cardiocirculatory and neurologic status, and the findings were recorded in the EHR. Blood samples for baseline analysis (values at admission) were collected within 30 min after the patient’s admission to the ICU. Subsequent blood samples were taken at least once daily, between 6:30 and 7:00 h, or more frequently at the discretion of the clinician. Laboratory analysis included the following parameters: CRP, PCT, hsTnI, NT-proBNP, lactate, and arterial blood pH. Additional data were also collected, such as information on targeted temperature management (TTM), hypotension, time from ROSC to admission to the ICU, and time from onset of cardiac arrest to ROSC. BLS efficiency was assessed by EMS members upon arrival at the scene. BLS was deemed efficient when the resuscitator was a trained first responder. If the resuscitator was a layperson, the evaluation included an assessment of the position of the hands, rate, depth, and technique of chest compression. Dedicated feedback devices were not utilized in the evaluation of BLS efficiency. Hypotension was defined as mean arterial pressure (MAP) less than 90 mmHg for at least 5 min. ST-elevation myocardial infarction (STEMI) was defined as new ST elevation at the J-point of at least 1 mm in two contiguous leads, except in V2–3, or in leads V2–3 of at least 2 mm in men and at least 1.5 mm in women. Heart failure was classified in accordance with the Killip–Kimball classification; class I included individuals with no clinical signs of heart failure, class II individuals with rales or crackles in the lungs, class III individuals with pulmonary edema, and class IV individuals in cardiogenic shock [24]. Comorbidities were considered present if the patient had at least one of the following: arterial hypertension, ischemic heart (coronary) disease, diabetes mellitus, dyslipidemia, malignancy (past or active), or cerebrovascular insult, regardless of whether it was known (and treated) prior to the cardiac arrest or not.

The data were analyzed using IBM SPSS Statistics for Macintosh (version 29.0.0.0 (241), IBM Corp., Armonk, NY, USA, 2022). We assessed the normality of samples in every analysis through a manual check of skewness and kurtosis and employed Shapiro–Wilk test for normality when the status was unclear. Descriptive statistics are presented as counts, percentages, means ± standard deviation (SD), or median with 95% confidence interval (CI) as appropriate. Differences between groups were analyzed with an independent *t*-test (parametric), Mann–Whitney U test (nonparametric), and chi-square or Fisher’s exact test (categorical). Tests with calculated two-tailed *p*-values of ≤0.05 were considered significant.

The calculated minimal sample size for the primary outcome (maximum NSE levels) to achieve a statistical power of 0.8 at a significance level of 0.05 was determined to be 44 patients (22 in each group). Similar sample sizes were calculated for most of the other outcomes.

## 3. Results

### 3.1. Baseline Data

**Figure 1:**
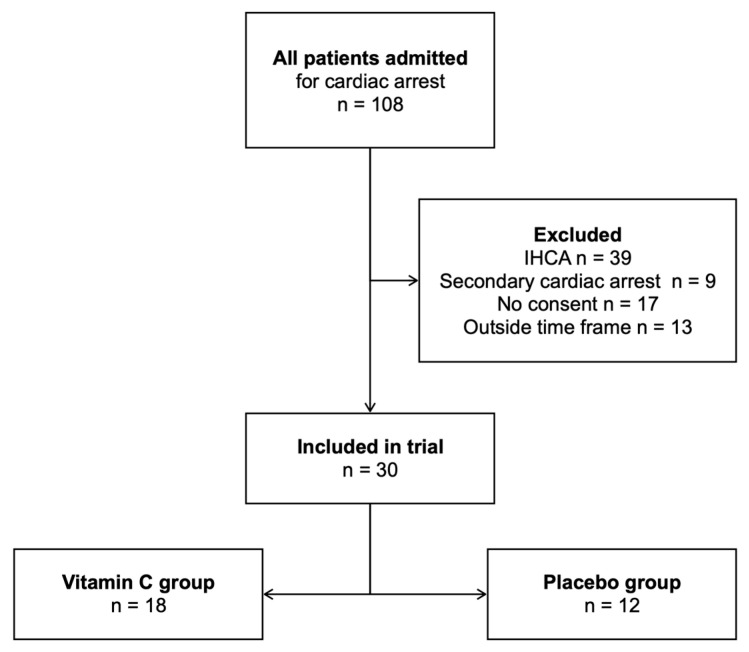
Inclusion and exclusion flowchart (IHCA, in-hospital cardiac arrest).

During the observed period, 108 patients were admitted to our ICU after cardiac arrest. After applying exclusion criteria, 30 patients were included in the trial and subsequent analysis: 18 (60%) in the intervention group (IG) that received vitamin C and 12 (40%) in the control group (CG) that received the usual standard of care. None of the included patients were lost to follow-up (e.g., due to transfer to another facility). Figure 1 illustrates the inclusion and exclusion of patients.

The average Sequential Organ Failure Assessment (SOFA) score of all patients at admission was 9.9 ± 2.4 and was lower in the IG compared to the CG. However, it is important to highlight that, given the comatose and mechanically ventilated state of all included patients upon admission, each patient received 7–8 points solely based on this criterion. Patients in the IG were, on average, slightly younger and had a higher body mass index (BMI). Two-thirds of patients in both groups were male, and most of all patients had some comorbidities, but the differences were not statistically significant. Based on these data, we can assume that the two groups were comparable. Table 1 presents basic demographic and prehospital data.

The majority of patients suffered cardiac arrest in front of witnesses, mostly at home or in public spaces. The IG had more instances of witnessed cardiac arrests. Over half of all victims received sufficient BLS measures, and in 23% of patients, an AED was used. However, owing to discrepancies in EMS records and the absence of a standardized nationwide OHCA registry, a lot of data about BLS and AED use are either missing or deliberately marked as such to prevent misinterpretation. The initial rhythm was shockable in half of all patients, with more instances in the IG. On average, the time from cardiac arrest to ROSC was 21.7 ± 8.6 min, with no significant difference between groups. Slightly more than one-third of all patients exhibited signs of ST-elevation myocardial infarction after ROSC, more in the IG. Endotracheal intubation during cardiac arrest was the method of choice for prehospital airway management in most cases.

### 3.2. Main Results

**Figure 2:**
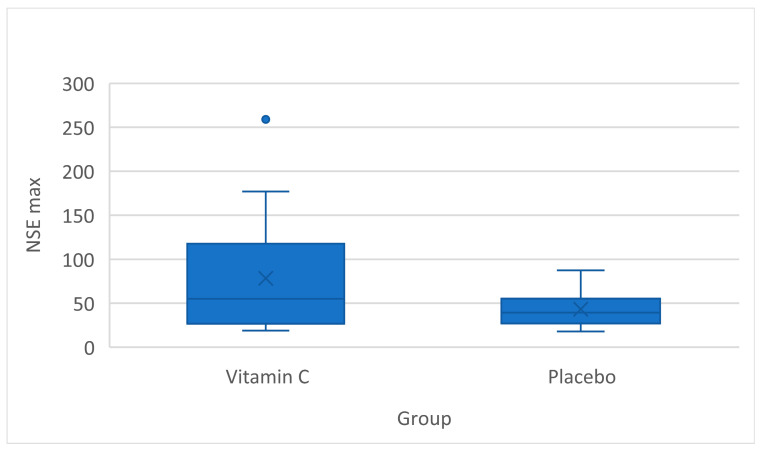
Comparison of maximum NSE levels between groups (NSE, neuron-specific enolase). Blue dot represents an extreme value, indicating the maximum NSE level recorded at 259 µg/L in a single patient.

The primary outcome was the maximum NSE serum level after the 96 h intervention, which was higher in the IG (55.05 µg/L [95% CI 26.7–124.0]) compared to the CG (39.4 µg/L [95% CI 22.6–61.9]), although the difference was not statistically significant (Figure 2).

Brain CT was conducted in the majority of all patients, with the initial result described as normal in most cases in both groups. Myoclonus in the first 72 h after cardiac arrest was observed in 27% of all patients, more in the IG. No significant differences were noted between the groups. Further details are presented in Table 2.

### 3.3. Other Outcomes

Table 3 summarizes relevant clinical data regarding the management of patients in the ICU, while Table 4 shows a comparison of laboratory values between groups.

Patients in the IG were mechanically ventilated for a shorter duration and were extubated more frequently compared to the CG. The majority of patients underwent TTM, mostly hypothermia (53%), with no significant differences between groups. Almost half of all patients experienced some form of arrhythmia requiring treatment. Patients in the IG had more ventricular arrhythmias, fewer supraventricular arrhythmias, and were without arrhythmias at all compared to the CG, with these differences being statistically significant (*p* = 0.044). Also, patients in the IG had noticeably fewer cases of new-onset heart failure. All patients with STEMI, regardless of the group, received PCI. About 20% of all patients had some infection already before cardiac arrest, while two-thirds of patients developed new infections after ICU admission. The differences between groups were not statistically significant. The median time from ROSC to ICU admission was higher in the IG. Patients in the IG spent significantly less time in the ICU; however, the duration of hospital stay was longer for patients in the IG. Overall, half of the patients survived at least 30 days after cardiac arrest, with more in the IG. No significant differences were observed regarding CPC upon discharge between groups.

Patients in both groups had negative baseline CRP and PCT. However, when comparing maximum values of these two parameters, patients in the IG had lower maximum CRP as well as PCT, with the difference in PCT being statistically significant. The same direction of differences between groups applies to other laboratory parameters as well, although not statistically significant. Patients in the IG had a higher baseline but lower maximal hsTnI, as well as lower maximal NT-proBNP and lactate, and a higher minimal pH of arterial blood.

## 4. Discussion

Our research showed that the maximum NSE serum level was higher in patients who received vitamin C compared to the placebo, even though the difference was not statistically significant. Furthermore, patients who received vitamin C had a higher percentage of myoclonus in the first 72 h. Based on these data, one can assume that patients with vitamin C were potentially at risk of developing more severe neurologic injury. However, current theoretical rationale, as well as results from animal and human studies, suggested that the antioxidizing effect of vitamin C could, among other effects, mitigate post-resuscitative neurological injury. Therefore, NSE levels should be lower in patients who received vitamin C.

The potential beneficial effects of intravenous vitamin C supplementation in critically ill patients have been predominantly studied in those with sepsis. Marik et al. demonstrated significantly lower mortality in patients, treated with early intravenous vitamin C, hydrocortisone, and thiamine, as well as more vasopressor-free days in the treatment group [25]. Several subsequent trials produced inconclusive results [26,27,28,29], all of which were conducted on patients with sepsis. However, sepsis and PCAS share similar pathophysiologic pathways [30,31,32]. Therefore, intravenous vitamin C is expected to yield similar results in post-cardiac arrest victims. We hypothesized that intravenous vitamin C would provide an antioxidizing effect and thus mitigate oxidative and nitrosative stress caused by free radicals after ischemic-reperfusion injury.

The brain’s vulnerability to ischemia, attributed to its high oxygen consumption and inability to store energy, particularly affects vital regions, responsible for sensory and motor functions, memory processing, cognition, and arousal [5,33,34,35,36]. Primary brain injury ensues from the abrupt cessation of cerebral blood flow, leading to the depletion of adenosine triphosphate (ATP) and subsequent neuron depolarization. This triggers a cascade of processes, including increased intracellular calcium concentration, which, among other effects, promotes the overproduction of superoxide and nitric oxide, facilitating the formation of peroxynitrite, a highly potent and reactive molecule that further exacerbates cellular damage and oxidative stress [37,38,39,40,41,42]. Ischemia also rapidly depletes intracellular antioxidants, including vitamin C [12,43]. Reperfusion amplifies these processes, escalating free radical production due to oxygen-rich blood, proinflammatory cytokine release, microthrombosis, and cerebrovascular dysfunction, further intensifying the initial tissue damage [12,44,45,46,47].

One of vitamin C’s key functions is acting as an antioxidant, where it donates a single electron to superoxide, hydroxyl, and other radicals, undergoing oxidation to form dehydroascorbic acid in the process [11,13]. This antioxidative activity is crucial for neutralizing harmful free radicals and safeguarding cells from oxidative stress. Humans, lacking the ability to synthesize vitamin C, must obtain it through their diet. Enteral uptake of vitamin C is dependent on sodium-dependent vitamin C transporter (SVCT) type 1 proteins, limited by saturation, preventing higher plasma concentrations [11,13,21]. In contrast, intravenous administration allows for supraphysiological concentrations of vitamin C. Brain capillary endothelial cells lack expression of SVCT type 2, a pivotal protein for ascorbate transport [11]. As a result, the primary pathway for vitamin C entry into the central nervous system (CNS) involves a two-step mechanism: initially, vitamin C is transported from the plasma to choroid plexus cells via SVCT2; then, it is subsequently released into the cerebrospinal fluid (CSF) through an unidentified mechanism and, finally, enters neurons once again through SVCT2 [12,13,48].

The beneficial effect of intravenous vitamin C should be most notably observed in neurons because they contain fewer endogenous antioxidants than other tissues [49,50]. The positive effects of vitamin C should be apparent with different levels of NSE, an established marker of brain injury. NSE is a γ-γ isoform of an enzyme enolase, which occurs only in neurons, and the extracellular concentration of NSE is detectable only when it is passively released from injured neurons [22,23].

However, in our study, 30-day survival was also higher in patients who received vitamin C, raising questions about the impact of initial severity of neurologic injury on later survival and functional outcomes. One possible explanation for this discrepancy could be that in patients treated with TTM, the reliability of early neuroprognostication is low [44]. Additionally, the higher 30-day survival in patients receiving vitamin C corresponds with a lower SOFA score upon admission.

Given the complexity of post-cardiac arrest pathophysiology and the systemic antioxidizing effect of intravenous vitamin C, we observed other clinical outcomes to establish a possible effect on other organ systems. Our findings regarding other clinical outcomes were more conclusive than those regarding neurological injury. Patients receiving vitamin C spent less time mechanically ventilated and were extubated at a higher percentage. Similar results were produced in other trials; however, the patient population and regimen were different from ours [51]. One study utilized the exact dosing regimen, albeit in patients with sepsis, in which the duration of mechanical ventilation was longer in patients that received vitamin C [51].

Slightly less than half of our patients developed arrhythmias that required treatment either with pharmacologic agents or electricity. Our results suggest a protective role of vitamin C for cardiac myocytes, as patients who received the placebo had significantly more arrhythmias that needed treatment. Findings from some other studies, though on different populations, also confirms the beneficial effect [52,53,54].

In our study, patients treated with intravenous vitamin C had a significantly shorter length of ICU stay, while the length of stay in the hospital was longer compared to patients who received the placebo. The longer hospital stay in the IG was likely attributed to the cause of cardiac arrest. Specifically, patients with STEMI remained in our hospital for additional cardiologic workup, whereas patients without STEMI did not require such extensive workup and were discharged earlier, even though their stay in the ICU was longer. Results from similar trials produced mixed outcomes [55]. Marik et al. [25] found no significant difference in length of stay in the ICU, the same as Sevransky et al. [26], who also found that patients who received vitamin C had a longer length of stay in the hospital, with a median of 1 day in both instances, although without a significant difference. Emadi et al. and Hu et al. found the same difference in shorter ICU and hospital lengths of stay for vitamin C, with similar significance of mean differences in days [52,53]. Regardless, patients discharged from the ICU sooner were likely to become clinically stable enough to be transferred earlier compared to patients with placebo.

Patients who received vitamin C had an insignificantly lower rate of infection after ICU admission. This aligns with theoretical considerations that vitamin C mitigates systemic inflammatory response syndrome (SIRS) following cardiac arrest. Specifically, oxygenated blood after the onset of reperfusion strengthens free radical production, while circulating proinflammatory cytokines promote the development of SIRS [4,46]. Our patients receiving vitamin C also had lower maximal values of CRP and PCT, with the difference in maximal PCT values being statistically significant. However, other similar trials produced insignificant differences, and Lamontagne et al. even demonstrated a higher risk of organ dysfunction in recipients of vitamin C [29].

The number of enrolled patients in our study was approximately the same as the number of cardiac arrests victims treated at our ICU in previous years [56]. On average, our patients were slightly older than the European average (71.6 vs. 67.6 years); however, they were almost universally comparable to the findings in the EuReCa Two study in terms of sex, location of cardiac arrest, witnesses, and BLS measures. Notably, our population had a higher percentage of an initial shockable rhythm (51% vs. 20%), which can be attributed to the fact that our population was already admitted to the ICU, whereas the population in the EuReCa study also included those without ROSC. The average SOFA score upon admission corresponds with similar studies [57,58]. Moreover, our SOFA score upon admission was lower in patients receiving vitamin C, who had higher 30-day survival, and these findings are consistent with other studies [57,58].

Our study faced several limitations that need to be explained in the context of mixed results. Firstly, it was a single-center trial with a small sample size, resulting in low statistical power. External factors, such as reduced rates of ROSC and ICU admissions during the pandemic, and a general decline in willingness (of lawful relatives) to participate in medical trials, influenced participant recruitment. Secondly, the initial levels of NSE were unknown, and subsequent samples were not necessarily obtained at the exact same time. Thirdly, the SOFA score was calculated only upon admission, limiting insights into illness severity during treatment. Additionally, the intervention group had a lower SOFA score at admission, which could contribute to bias in favor of vitamin C. However, it is important to note that the mean difference in SOFA score was 1.9, and each included patient received the majority of points solely for being comatose and mechanically ventilated at admission. Fourthly, management decisions for individual patients were at the clinicians’ discretion, leading to non-uniform procedures. Furthermore, some data for certain variables are incomplete and missing, preventing significant conclusions.

Still, our study acknowledges these limitations and emphasizes the need for cautious interpretation. External factors impacted participant recruitment, with eligible patients showing reduced willingness to participate. Nevertheless, demographic and clinical similarities, including comparable duration of cardiac arrest, support meaningful comparison between groups.

## 5. Conclusions

We aimed to explore the promising possibility of treating PCAS in adult OHCA survivors with intravenous vitamin C. The results of our research were not conclusive in confirming beneficial effects. On one hand, vitamin C worsened surrogates of neurological injury and on the other, it improved markers of inflammation and parameters of myocardial injury. Given the mixed results, intravenous vitamin C should not be used outside of clinical trials. Further research is warranted.

## Figures and Tables

**Table 1:** Basic demographic and prehospital data.

	All (n = 30)	Intervention Group (n = 18)	Control Group (n = 12)	*p*
SOFA score at admission mean ± SD	9.9 ± 2.4	9.2 ± 2.6	11.1 ± 1.7	0.049
Age mean ± SD	71.6 ± 8.5	70.4 ± 8.2	73.3 ± 9.1	ns
BMI [kg/m^2^] mean ± SD	27.0 ± 3.1	27.4 ± 3.5	26.4 ± 2.2	ns
Gender				ns
male, n (%)	20 (67%)	12 (67%)	8 (67%)	
female, n (%)	10 (33%)	6 (33%)	4 (33%)	
Comorbidities, n (%)	23 (77%)	14 (78%)	9 (75%)	ns
Witnesses of cardiac arrest				ns
laypersons, n (%)	21 (70%)	12 (66%)	9 (75%)	
EMS, n (%)	3 (10%)	3 (17%)	0	
none, n (%)	6 (20%)	3 (17%)	3 (25%)	
Location of cardiac arrest				ns
home, n (%)	11 (37%)	8 (44%)	3 (25%)	
work, n (%)	1 (3%)	1 (6%)	0	
road, n (%)	2 (7%)	2 (11%)	0	
public area, n (%)	8 (27%)	4 (22%)	4 (33%)	
other, n (%)	3 (10%)	1 (6%)	2 (17%)	
no data, n (%)	5 (16%)	2 (11%)	3 (25%)	
Presumed medical cause, n (%)	30 (100%)	18 (100%)	12 (100%)	ns
BLS by witnesses				ns
sufficient chest compression, n (%)	13 (43%)	9 (50%)	4 (33%)	
sufficient chest compression and respirations, n (%)	6 (20%)	3 (17%)	3 (25%)	
no data, n (%)	11 (37%)	6 (33%)	5 (42%)	
AED use				ns
shock delivered, n (%)	4 (13%)	3 (17%)	1 (8%)	
shock not delivered, n (%)	3 (10%)	1 (6%)	2 (17%)	
available but not used, n (%)	2 (7%)	2 (11%)	0	
unavailable, n (%)	7 (23%)	4 (22%)	3 (25%)	
no data, n (%)	14 (47%)	8 (44%)	6 (50%)	
Initial rhythm				ns
shockable, n (%)	15 (50%)	11 (61%)	4 (33%)	
non-shockable, n (%)	11 (37%)	6 (33%)	5 (41%)	
no data, n (%)	4 (13%)	1 (6%)	3 (25%)	
Time to ROSC [min.] mean ± SD	21.7 ± 8.6	21.0 ± 8.8	22.6 ± 8.7	ns
STEMI after ROSC				ns
yes, n (%)	11 (37%)	8 (44%)	3 (25%)	
no, n (%)	15 (50%)	9 (30%)	6 (20%)	
no data, n (%)	4 (13%)	1 (6%)	3 (25%)	
Prehospital airway management				ns
supraglottic only, n (%)	3 (10%)	1 (6%)	2 (17%)	
supraglottic followed by ETI, n (%)	2 (7%)	2 (11%)	0	
ETI, n (%)	22 (73%)	14 (78%)	8 (67%)	
surgical, n (%)	0	0	0	
no data, n (%)	3 (10%)	1 (6%)	2 (17%)	

Abbreviations: AED, automated external defibrillator; BLS, basic life support; BMI, body mass index; EMS, emergency medical services; ETI, endotracheal intubation; min., minutes; ns, non-significant; ROSC, return of spontaneous circulation; SD, standard deviation; SOFA, sequential organ failure assessment; STEMI, ST-elevation myocardial infarction.

**Table 2:** Primary and secondary outcomes regarding neuroprognostication.

	All (n = 30)	Intervention Group (n = 18)	Control Group (n = 12)	*p*
NSE maximum level [µg/L]	39.4	55.05	39.4	ns
median [95% CI]	[31.3–79.0]	[26.7–124.0]	[22.6–61.9]	
CT first result				ns
normal, n (%)	15 (50%)	10 (56%)	5 (42%)	
slightly abnormal, n (%)	6 (20%)	4 (22%)	2 (17%)	
moderately abnormal, n (%)	1 (3%)	1 (6%)	0	
severely abnormal, n (%)	1 (3%)	0	1 (8%)	
not performed, n (%)	7 (23%)	3 (17%)	4 (33%)	
Myoclonus in first 72 h				ns
yes, n (%)	8 (27%)	6 (33%)	2 (17%)	
no, n (%)	12 (40%)	8 (44%)	4 (33%)	
no data, n (%)	10 (33%)	4 (22%)	6 (50%)	

Abbreviations: CI, confidence interval; CT, computed tomography; EEG, electroencephalogram; ns, non-specific; NSE, neuron-specific enolase.

**Table 3:** Clinical data during intensive care unit stay.

	All (n = 30)	Intervention Group (n = 18)	Control Group (n = 12)	*p*
Mechanical ventilation * [h]	135 [81–276]	92 [60–432]	187 [82–276]	ns
Extubation, n (%)	9 (30%)	6 (33%)	3 (25%)	ns
TTM				ns
hypothermia, n (%)	16 (53%)	9 (50%)	7 (58%)	
normothermia, n (%)	7 (23%)	4 (22%)	3 (25%)	
yes, but no temperature noted, n (%)	2 (7%)	1 (6%)	1 (8%)	
no, n (%)	2 (7%)	1 (6%)	1 (8%)	
no data, n (%)	3 (10%)	2 (11%)	1 (8%)	
Hypotension, n (%)	26 (87%)	17 (94%)	9 (75%)	ns
Arrhythmias				0.044
supraventricular, n (%)	6 (20%)	1 (6%)	5 (42%)	
ventricular, n (%)	7 (23%)	5 (28%)	2 (17%)	
both, n (%)	1 (3%)	0	1 (8%)	
none, n (%)	10 (33%)	8 (44%)	2 (17%)	
no data, n (%)	6 (20%)	4 (22%)	2 (17%)	
Heart failure				ns
Killip I, n (%)	5 (17%)	3 (17%)	2 (17%)	
Killip II, n (%)	4 (13%)	1 (6%)	3 (25%)	
Killip III, n (%)	2 (7%)	1 (6%)	1 (8%)	
Killip IV, n (%)	2 (67%)	0	2 (17%)	
none, n (%)	15 (50%)	12 (67%)	3 (25%)	
no data, n (%)	2 (7%)	1 (6%)	1 (8%)	
PCI, n (%)	11 (37%)	8 (44%)	3 (25%)	ns
Renal replacement therapy, n (%)	3 (10%)	2 (11%)	1 (8%)	ns
Infection				ns
before cardiac arrest, n (%)	6 (20%)	5 (28%)	1 (8%)	
VAP, n (%)	3 (10%)	2 (11%)	1 (8%)	
sepsis, n (%)	9 (30%)	4 (22%)	5 (42%)	
other, n (%)	8 (27%)	5 (28%)	3 (25%)	
no data, n (%)	4 (13%)	2 (11%)	2 (17%)	
Time ROSC to admission * [min]	127 [60–170]	130 [60–266]	95 [24–178]	ns
Time spent in ICU * [h]	175 [116–309]	122 [78–193]	210 [154–436]	0.031
Time spent in hospital * [days]	8.6 [6.0–23.0]	14.9 [4.0–27.0]	8.0 [6.5–28.0]	ns
Survived				ns
30 days, n (%)	16 (53%)	11 (61%)	5 (42%)	
of those with CPC 1–2, n (%)	7 (44%)	5 (46%)	2 (40%)	
of those with CPC 3–4, n (%)	6 (34%)	3 (27%)	3 (60%)	
of those not specified, n (%)	3 (19%)	3 (27%)	0	

* values expressed as: median [95% confidence interval]. Abbreviations: CPC, cerebral performance category; ICU, intensive care unit; min., minutes; ROSC, return of spontaneous circulation; SD, standard deviation; SOFA, sequential organ failure assessment; ns, non-significant; TTM, targeted temperature management; PCI, percutaneous coronary intervention; VAP, ventilator-associated pneumonia.

**Table 4:** Comparison of laboratory values between groups.

	All * (n = 30)	Intervention Group * (n = 18)	Control Group * (n = 12)	*p*
CRP [mg/L]				ns
on admission	3 [3–7]	3 [3–7]	3 [3–14]	
minimum	3 [3–7]	3 [3–5]	3 [3–14]	
maximum	178 [154–258]	176 [150–258]	221 [154–453]	
PCT [µg/L]				
on admission	0.03 [0.03–0.13]	0.03 [0.03–0.23]	0.03 [0.03–0.43]	ns
minimum	0.13 [0.03–0.42]	0.10 [0.03–0.23]	0.42 [0.03–14.12]	ns
maximum	3.06 [0.69–10.76]	0.79 [0.52–7.46]	10.76 [1.84–98.32]	0.025
hsTnI [ng/L]				ns
on admission	441 [126–1502]	619 [84–4438]	441 [16–2295]	
minimum	317 [71–1502]	574 [71–2202]	295 [16–2295]	
maximum	10,993 [2621–20,261]	10,993 [968–21,976]	13,304 [706–84,093]	
NT-proBNP [pmol/L]				ns
on admission	167 [54–325]	167 [49–325]	2078 [54–4101]	
minimum	143 [78–469]	123 [57–325]	469 [78–873]	
maximum	484 [202–873]	355 [109–1143]	827 [134–1997]	
Lactate [mmol/L]				ns
on admission	5.3 [3.0–7.7]	4.6 [2.5–7.7]	6.1 [3.0–13.5]	
minimum	1.1 [0.8–1.3]	1.2 [0.9–1.4]	1.1 [0.6–1.3]	
maximum	6.1 [3.8–7.8]	4.9 [3.0–9.7]	6.3 [3.8–13.5]	
pH of arterial blood				ns
on admission	7.243 [7.170–7.296]	7.236 [7.170–7.296]	7.263 [6.929–7.313]	
minimum	7.236 [7.090–7.283]	7.236 [7.151–7.296]	7.216 [6.929–7.298]	
maximum	7.471 [7.449–7.488]	7.474 [7.459–7.493]	7.455 [7.441–7.488]	

* values expressed as: median (95% confidence interval). Abbreviations: NT-proBNP, N-terminal pro b-type natriuretic peptide; CRP, C-reactive protein; hsTnI, high-sensitivity troponin I; ns, non-significant; PCT, procalcitonin.

## Data Availability

The data used in the analysis are available from the corresponding author upon request.
